# Mitral valve thickening in acute rheumatic fever as a predictor of late valvar dysfunction

**DOI:** 10.1371/journal.pone.0259737

**Published:** 2021-11-17

**Authors:** Telêmaco Luis da Silva, Antonio Pazin-Filho, Minna M. D. Romano, Virgínia P. L. Ferriani, José A. Marin-Neto, Benedito C. Maciel, André Schmidt

**Affiliations:** 1 Internal Medicine Department, Medical School of Ribeirão Preto, University of São Paulo, São Paulo, Brazil; 2 Pediatrics Department, Medical School of Ribeirão Preto, University of São Paulo, São Paulo, Brazil; Kurume University School of Medicine, JAPAN

## Abstract

**Background:**

Rheumatic heart disease (RHD) complicating acute rheumatic fever (ARF) remains an important health problem in developing countries. No definitive diagnostic test for ARF exists and the role of Doppler echocardiography (DEC) for long-term prognostic evaluation following ARF is not well established.

**Objective:**

To investigate the prognostic value of DEC in patients with ARF as a predictor of chronic valve dysfunction.

**Methods:**

Prospectively enrolled patients with clinical ARF had a DEC performed soon after diagnosis and repeated at 1, 3, 6 and 12 months and thereafter at every 1–2 years. We defined chronic valve dysfunction by ≥ 3 of the following: increased valve thickening, commissure fusion, subvalvular thickening, reduced leaflet mobility, non-trivial mitral and/or aortic regurgitation. We performed univariate analysis and developed multivariate logistic regression models to identify variables that may influence evolution to RHD. p <0.05 was considered significant.

**Results:**

We evaluated 70(57% men) patients, 10.8±5.6 years-old during the ARF episode and followed for 95±26 months. Chronic valve dysfunction was identified in 36(51.4%) which fulfilled criteria for RHD and 10(27.8%) of them died or underwent valve surgery. Univariate analysis showed that mitral valve thickening and presence of mitral regurgitation at baseline DEC, were associated with RHD(p<0.01). Multivariate logistic regression showed that only mitral valve thickness either as a continuous (Odds-Ratio:5.8;95%CI:1.7–19.7) or as a categorical variable (Odds-Ratio:4.04;95%CI:1.06–15.3) was an independent predictor of chronic valve dysfunction.

**Conclusions:**

Mitral leaflets thickening documented at the time of diagnosis of ARF is a consistent prognostic marker for the subsequent evolution to RHD.

## Introduction

Acute rheumatic fever (ARF) is a systemic inflammatory immunologically mediated complication of a streptococcal upper respiratory infection [1]. It involves, in most cases, the heart, joints, central nervous system, subcutaneous tissue and skin. With the exception of the cardiac involvement, the other clinical manifestations do not determinate permanent organ dysfunction. Since the publication of what is now known as the “Jones criteria” in 1944, the basis for the clinical diagnosis of ARF was defined [2]. More recently, a revision of those criteria included echocardiographic features as part of diagnostic criteria [3], a concept previously suggested by the World Heart Federation [4].

The detection of echocardiographic signs of acute inflammatory changes in the absence of clinical signs of inflammation is now widely recognized as evidence of subclinical carditis, with a mean prevalence of 16.8% in scarse published series with insufficient follow-up data [3, 5, 6]. However, it seems relevant to focus on early detection of those morphological and functional changes showing acute carditis and correlate them with extended follow-up data to provide a confirmation of its participation in the development of Rheumatic Heart Disease (RHD).

Rheumatic heart disease involving the chronic sequelae of the inflammation in ARF, still represents a significant health system burden in most of the underdeveloped world with an endemic presentation in several countries [7]. It has gained some interest recently due to the possible use of echocardiography as a tool to improve early diagnosis of heart involvement, in an attempt to reduce its long-term morbidity and mortality [8–10]. Some recent publications identified mitral involvement as common in subclinical RHD [11], but the search for a specific test for ARF is still an open issue [12].

Due to limited availability of prospective studies reporting on the ARF echocardiographic findings, this investigation was designed to explore the diagnostic and prognostic value of serial echocardiography findings during ARF in the development of RHD in the long-term follow-up.

## Methods

We enrolled consecutive patients who had a first clinical diagnosis of ARF when evaluated at the Ribeirão Preto Medical School University Hospital, University of São Paulo, from June 1991 to November 1996. No patient reported any previous episode of ARF. All patients or their parents, if minors, provided written informed consent and the study was approved by the Institutional Review Board of the Ribeirão Preto Medical School University Hospital.

We used the 1992 modified Jones criteria to establish the clinical diagnosis of ARF [13]—two major criteria (carditis, polyarthritis, Sydenham chorea, *erithema marginatum* or subcutaneous nodules) or one major and two minor criteria (artralgia, fever, elevated erythrocyte sedimentation rate or C-reactive protein, first-degree AV block), and evidence of recent streptococcal infection. We excluded patients with previous diagnosis of RHD or other etiology for heart disease.

### Study protocol

We performed serial Doppler echocardiographic (DEC) examinations before any treatment, 24 to 48 hours after clinical diagnosis of ARF and 1, 3, 6 and 12 months thereafter. Then, we repeated DEC during follow-up yearly or every two years. All patients were admitted to the institution and received intravenous ou intramuscular antibiotics (mainly penicillin) as part of the medical treatment.

We performed Color Doppler echocardiography on Hewlett-Packard (Andover, Massachusetts) machines models Sonos, 1000, 2500 or 5500, with appropriate transducers and gain and pulse repetition frequencies registered in order to be reproduced in follow-up exams. Patients were positioned in left lateral decubitus and cine loop images on parasternal long and short (mitral valve, papillary muscles and apical levels) axis, as well as apical four-chamber, apical two-chamber and longitudinal apical views. Color Doppler, pulsed and continuous Doppler of all valves in each view were also collected. From each study the following features and parameters were actively searched and registered when present: valvular regurgitation (jet length, jet area and the relationship between jet area and receiving chamber area) valvular nodulations, chamber dimensions adjusted to body size, left ventricular ejection fraction (LVEF), leaflet thickness, mobility of leaflets, subvalvar apparatus involvement and pericardial fluid accumulation.

The study endpoint was the identification of chronic valvar dysfunction, defined as the concomitant presence of at least three of the following: leaflet thickening, commissural fusion, sub-valvar apparatus thickening, reduction of leaflets mobility and signs of non-physiologic mitral and/or aortic regurgitation. We considered valvar regurgitation as non-physiologic when two or more of the following criteria were present: a regurgitant jet in two or more view plans, persistence of the jet for > 50% of the cardiac cycle, a well-defined envelope in the spectral Doppler, receiving chamber jet with mosaic appearance or a well-defined proximal isovelocity image without baseline adjustments. For chronic involvement, a concordant opinion of two physicians with extensive echocardiographic experience was mandatory. Although a detailed description of the findings was included in the results report released for the clinical staff, no speculation of its value for the ARF diagnosis was mentioned as those findings were not defined as characteristic of this entity.

All images were analyzed off-line. We measured chamber dimensions, jet areas and LVEF (Simpsons`rule) according to recommendations of the American Society of Echocardiography and expressed as the mean value from three consecutive cardiac cycles for all individuals. All measurements were made by one experienced cardiologist with expertise in echocardiography (TLSJr) and revised by another (APF). If any disagreement occurred, a senior cardiologist (BCM) made the final decision.

We collected clinical data in a dedicated Access Database (Microsoft Corporation, USA), including age, weight, height, major (poliathritis, carditis, Sydenham chorea, *erythema marginatum* and subcutaneous nodules) and minor (arthralgia, fever, erythrocyte sedimentation rate, C-reactive protein and EKG prolonged PR interval) Jones criteria. We also documented previous history of ARF episode, previous RHD diagnosis, family history of ARF/RHD, clinical or radiological signs of heart failure, heart murmurs or pericardial friction rub inflammatory markers and anti-streptolysin-O titers. We collected information regarding duration and type of clinical treatment received from hospital notes during the protocol, as well as surgeries and death reports.

### Statistical analysis

We summarized categorical variables as a percentage and quantitative variables as a mean and standard deviation or median (25 and 75 percentiles) accordingly. To compare categorical variables, we used the Fisher test or the Chi-Square test for trend and independence as appropriate. We used Student’s t-test and non-parametric equivalent (Mann-Whitney) or analysis of variance and nonparametric equivalent (Kruskal-Wallis) to compare continuous variables.

Considering the evolution outcome for RHD according to clinical, surgical or DEC criteria, we obtained Receiver Operating Curves (ROC) for the echocardiographic variables analyzed. From the stipulated curves, we used the value of the best relationship between sensitivity and specificity to create binary variables above and below the limit obtained for the construction of logistic regression models.

We developed multiple logistic regression models to calculate the Odds Ratio (OR) of several variables that may influence evolution to RHD. We evaluated the association of echocardiographic variables and the outcome independently of confounding factors. All models included demographic variables (gender and age), presence or absence of clinically defined rheumatic carditis, changes in electrocardiogram and chest radiography compatible with ARF, and presence and severity of valvular (aortic and mitral) dysfunction corrected by the left atrium area or the left ventricular outflow tract. The models varied only in the way of expressing the echocardiographic Doppler variables. Model I included continuous variables, being arbitrarily assigned zero value in cases where mitral or aortic regurgitation was absent. Model II included the variables in the binary form, using the best value between sensitivity and specificity (obtained in the ROC analysis) as the limit. We used the Wald test to evaluate the significance of each model.

For both models, we used incremental modelling, parting from the exposure (echocardiographic variables) and the outcome and including progressive clinical variables. We used this approach to evaluate the presence of collinearity among the variables and we performed modelling for the impact of the interaction of two associate variables.

For all the tests used, the value of p <0.05 was considered statistically significant.

We used STATA Intercool version 9.2 for statistical analysis and graph construction.

## Results

Seventy ARF patients, 40(57%) males, between 3 and 40 years-old (10.8 ± 5.6), were included. Mean follow-up was 95 ± 26 months varying from 88 to 101 months. Thirty-four patients (48.6%) did not develop RHD during follow-up–Group I, and 36 patients did–Group II. Follow-up duration was similar between groups (Group I: 92.3 ± 25.4 months; Group II: 97.3 ± 26.8 months; p = 0.42).

Ten patients from Group II developed severe RHD with 3 deaths and 7 underwent a surgical valve procedure during follow-up. Surgery was performed after 2408 ± 2002 days following the acute event either as mitral valve repair (3 patients) or valvar replacement. Two deaths were due to severe heart failure several years after the acute rheumatic fever event (7 and 11 years later) and one patient presented a sudden cardiac death event (11 years after the first episode of acute rheumatic fever) with no autopsy report available.

Table 1 presents anthropometric, clinical and laboratorial variables at baseline, including major Jones criteria for the two groups according to development of RHD at follow-up. Those who developed chronic valve dysfunction had more clinical and laboratorial signs of carditis at baseline, such as heart murmurs, enlarged hearts at x-ray, and abnormal EKGs. Polyarthritis was the most frequent major Jones criterion (55.7%), followed by carditis (24.3%). Carditis incidence was significantly distinct between groups (Group I = 8.8% vs Group II = 38.9%; p = 0.001). Similarly, enlarged heart silhouette at chest x-ray occurred only in patients from Group II, although an abnormal EKG (first degree atrioventricular block) was noted in a smaller proportion (23.5%) in Group I compared to Group II (66.6%; p<0.01).

**Table 1:** Anthropometric, Jones criteria, clinical and laboratorial variables in according to the presence (Group II) or absence (Group I) of Rheumatic Heart Disease at the end of the follow-up.

		GROUP I	GROUP II	p
	Number (%)	34 (48.6)	36 (51.4)	
	Age in years—median(25%-75% IQ)	10 (8–12)	10 (7.5–11)	0.705
	Male gender (%)	20(58.8)	20(55.6)	0.782
**History**				
	Family history of RF(%)	1(3.2)	6(17.1)	0.067
	Fever (%)	26(78.8)	22(61.1)	0.111
	Arthritis (%)	19(55.9)	20(55.5)	0.978
	Carditis(%)	3(8.8)	14(38.9)	0.001
	Sydenhan Chorea (%)	1(2.9)	8(22.8)	0.014
	Subcutaneous nodules (%)	1(3.0)	1(2.8)	0.950
	Erithema marginatum (%)	1(2.9)	2(5.5)	0.589
	Artralgia(%)	22(66.6)	13(36.1)	0.011
**Physical Examination**				
	Heart Failure(%)	0(0)	7(20.0)	
	Pericardial rub(%)	0(0)	0(0)	
	Heart murmur(%)	20(58.8)	31(86.1)	0.010
	Sydenhan Chorea (%)	1(2.9)	7(19.4)	0.030
	Subcutaneous nodules (%)	1(2.9)	1(2.8)	0.967
**Laboratorial findings**				
	Abnormal EKG(%)	8(23.5)	24(66.6)	<0.01
	Abnormal chest X rays (%)	0(0)	10(31.2)	
	Abnormal hemogram (%)	24(70.6)	27(75.0)	0.678
	Positive CRP (%)	25(78.1)	18(51.4)	0.023
	ESR (%)	24(70.6)	23(63.9)	0.551
**Treatment**				
	Aspirin (%)	15(44.1)	13(16.1)	0.494
	Corticosteroids (%)	22(64.7)	28(77.8)	0.226
**Outcome**				
	Surgery (%)	0(0)	9(0)	
	Death(%)	0(0)	3(8.3)	

CRP: C reactive protein; EKG: Electrocardiogram;ESR: Erithrocyte sedimentation rate.

Table 2 summarizes echocardiographic variables according to the later development of RHD. By univariate analysis, mitral valve leaflets thickening was significantly distinct between groups, indicating a more prominent involvement at baseline in Group II. Left cardiac chambers were also significantly larger in those who later developed RHD (Group II). Finally, mitral regurgitation was documented at baseline examination in 59 (84.3%) of the 70 patients of this sample, this abnormality occurring in 34 out of the 36 patients from group II compared to 25 out of 34 belonging to Group I (Chi-square test = 0.016). In contrast aortic regurgitation was identified in only 28 cases at baseline (14 in each group) and no difference between groups was observed (p = 0.845). Several mitral regurgitation indicators of severity such as regurgitant fraction, mitral jet area and relation between regurgitant jet area and left atrium area, were significantly more severe in Group II at baseline examination. For aortic regurgitation, only regurgitant jet width was significantly different between the two groups at baseline examination.

**Table 2:** Echocardiographic parameters obtained at baseline examination and after 6 months, expressed as median (25th and 75th percentiles).

	GROUP I		GROUP II			P-Value		
PARAMETER	Baseline	6 months	Baseline	6 months	Baseline Group I vs Group II	6 months Group I vs Group II	Baseline Group I vs 6 months	Baseline Group II vs 6 months
Aorta dimension (mm)	25(21;26)	23.5(22;28)	23(22;26)	24(21.5;25.5)	0.787	0.627	0.801	0.833
LA dimension (mm)	30(28;34)	29(27;33)	37(30;44)	31(28.5;37.5)	0.001	0.072	0.146	0.044
RV dimension (mm)	15(13;14)	16(15;16)	16(14;17)	17(14;18)	0.544	0.893	0.279	0.255
LVEDD dimension (mm)	44.5(40;50)	43(39;50)	48.5(43.5;52.2)	46(42;52)	0.020	0.063	0.357	0.196
LVESD dimension (mm)	26(23;31)	27(24;30)	27.5(26;31)	29(25.5;30.5)	0.089	0.174	0.984	0.591
IVSD dimension (mm)	6(5;7)	6(5;7)	6(6;7)	6(5.5;7)	0.568	0.703	0.906	0.941
LVOT dimension (mm)	16(14;17)	17(16;22)	18(16;20)	18.5(16;21)	0.371	0.778	0.782	0.369
Mitral valve thickness (mm)	3.2(2.8;3.4)	3.2(2.8;3.3)	3.9(3.3;5.25)	3.4(3.25;5)	<0.01	<0.01	0.580	0.315
Aortic valve thickness (mm)	2.8(2.5;3.2)	2.8(2.7;2.9)	2.8(2.6;3.4)	2.9(2.8;3.2	0.251	0.305	0.707	0.782
Mitral Regurgitation (%)	25(73.3)	10(30.3)	34(94.4)	22(68.7)	0.016	0.002	0.001	0.004
Aortic Regurgitation (%)	14(41.2)	3(9.1)	14(38.9)	8(25.0)	0.845	0.087	0.003	0.221
Mitral regurgitant jet area (cm2)	5(3.5;6.4)	4.4(3.7;5)	7.5(6;12)	6.9(4.9;8.8)	<0.01	0.027	0.563	0.471
Aortic regurgitant jet area (cm2)	1.1(0.7;1.2)	1.3(0.7;2.1)	1.6(0.9;2.8)	1.4(0.9;2.5)	0.047	0.642	0.671	0.449
Aortic regurgitant jet width (mm)	5.5(4;6.5)	5.5(4.5;7.4)	7(6;10)	7(6;9)	<0.01	0.280	0.490	0.729
Mitral jet area/LA area (%)	26(21.4;44)	26(20;29)	48.3(36.8;68)	41.5(30.5;52)	<0.01	0.018	0.399	0.121
Aortic jet area/LVOT area (%)	20(17;32)	25.8(15.2;36.8)	32.5(17.6;71)	26.3(13.8;50)	0.160	0.877	0.915	0.377

Values are shown with statistical differences between Group I (individuals who did not developed Rheumatic Heart Disease) and Group II (those who developed Rheumatic Heart Disease) at distinct timelines.

LA–Left Atrium; LVEDD—Left ventricle end diastolic dimension; LVESD–Left ventricle end systolic dimension; RV–Right ventricle; IVS–interventricular septum dimension; LVOT–Left ventricle outflow tract.

In the long-term evaluation at 6 months, only parameters related to mitral valve involvement, such as leaflet thickness and regurgitant jet parameters had statistically distinct values from baseline values that differentiated the two groups—Table 2. Baseline median thickness of mitral valve leaflets was significantly higher in Group II (3.9–3.3; 5.25) patients at baseline compared to Group I patients (3.2–2.8; 3.4; p< 0.01), and the difference was still significant (p<0.01) at 6 months follow-up between the two groups.

**Fig 1:**
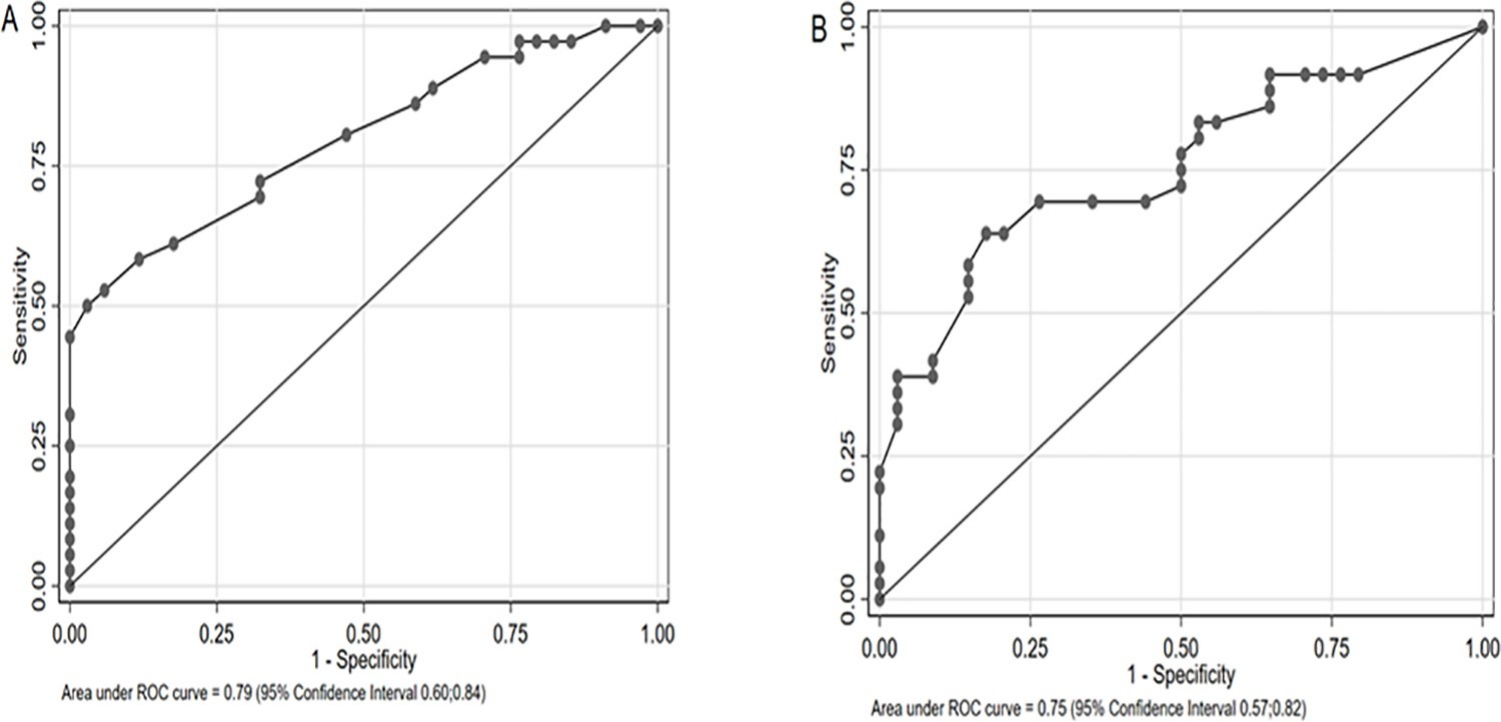
Receiver Operating Curve for (A) mitral valve leaflets thickness (mm) and (B) mitral regurgitation jet area (cm^2^) at baseline examination and development of RHD at follow-up.

A receiver-operating curve (ROC), considering the value obtained by manual measurement of the mitral valve leaflets thickness was constructed based on the later development of RHD with an area under curve of 0.79 (95% CI 0.6;0.84)–Fig 1A. Based on this analysis, we selected a mitral thickness leaflet of 3.3mm as a cut-off for our Model 2 for multivariate analysis. This value had a 72.2% sensitivity, 67.6% specificity, 70% correct classification, 2.23 positive likelihood ratio and 0.41 negative likelihood ratio for the outcome. Similarly mitral regurgitation jet area had a ROC area of 0.70 (95% CI 0.57; 0.82)–Fig 1B. A cut-off for mitral regurgitation jet area of 6.0 cm^2^ had a 69.4% sensitivity, 73.5% specificity, 71.4% correct classification, 1.2 positive likelihood ratio and 0.7 negative likelihood ratio in regard to the outcome. This was the cut-off value selected for Model 2 for multivariate analysis.

**Fig 2:**
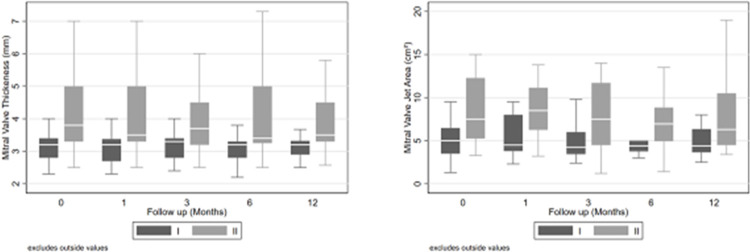
Box-plots of mitral valve leaflets thickness (A) and mitral regurgitant jet area (B) at baseline examination (0) and during the first follow-up year showing that abnormal changes identified at baseline in Groups I (not developed RHD) and II (who developed RHD) persisted during the first year after the ARF episode.

The severity of mitral regurgitation expressed by echocardiographic parameters and mitral valve leaflets thickness at baseline examination were significantly higher in Group II patients who later developed RHD. This difference persisted in follow-up examinations during the first 12 months after baseline examination, carried out during the ARF episode–Fig 2.

Aortic valve thickness was similar between groups at baseline and at 6 months examinations. For aortic regurgitation measurements, jet area was marginally significant at baseline (p = 0.047) but lost significance at the 6-month exam. Aortic regurgitant jet was larger in Group II patients but also lost its statistical significance at 6 month examination, The relation between regurgitant jet area and LVOT area at baseline examination, although larger in Group II patients, did not reach statistical significance for the difference between the groups (p = 0.16)–Table 2.

### Multivariate analysis

Table 3 presents the final logistic regression models constructed based on the univariate analysis using mitral valve thickness and mitral regurgitation as continuous variables (Model I) or as categorical variables based on the ROC analysis (Model II). The final models did not include aortic valve thickness or aortic regurgitation since their odds ratios have shown negative effects to the development of the outcome, which could be a sign of collinearity. We also tried to include an interaction factor representing mitral valve thickness and regurgitation, but it was not significant.

**Table 3:** Odds Ratio (OR) and 95% confidence interval for the variables included in Models I and II.

	Model I*	Model II**
Age (years)	0.94(0.85;1.05)	0.96(0.86;1.07)
Male gender	1.8(0.5;6.2)	2.03(0.56;7.3)
Carditis	1.3(0.55;3.14)	1.2(0.5;2.9)
Mitral leaflets thickness	5.8(1.7;19.7)ψ	4.04(1.06;15.3) ψ
Mitral regurgitation	1.01(0.8;1.26)	3.6(0.97;12.9)

*Mitral valve thickness and regurgitation included as continuous variables.

**Mitral valve thickness and regurgitation included as categorical variables based on ROC analysis.

ψ Significant values.

## Discussion

Rheumatic heart disease still is a very prevalent condition worldwide, especially in underdeveloped countries and was defined as a priority by the World Health Organization [14]. Clinical diagnosis of ARF is challenging and the recent addition of echocardiographic criteria to compose modified Jones criteria has improved the characterization of carditis in its various manifestations, even those running a subclinical course [15]. Current epidemiological surveys are aimed to identify RHD in its latent early manifestations, allowing the identification of patients who need secondary prevention with long-acting penicillin and close medical follow-up to avoid late severe complications. With the development of handheld low cost sonographs epidemiological surveys are spreading [16].

Our study sample consisted of patients with clinical diagnosis of ARF, and echocardiographic results were descriptive, not suggesting the occurrence of carditis on the report since we collected data before those parameters being considered as signs of ARF. One of its major strengths was a long follow-up period and the use of hard events (death, valvar surgery and a strict chronic valvar dysfunction definition). We observed a solid and easily obtainable diagnostic and prognostic marker of evolution to RHD after univariate and multivariate analysis: mitral valve thickening, either as a continuous or as a categorical variable with a cut-off value of 3.3 mm. This contrasts with previous seminal studies that used only a visual classification to define leaflet thickening. Another strength of our study is that outcome definition was very strict (death, surgery or a composite of three echocardiographic signs).

Vasan et al. identified mitral valve thickening in all patients with previous ARF episodes and signs of carditis in a new ARF episode and in 40% of those presenting a first episode of ARF but with signs of carditis [17]. Lanna et al, evaluating 28 ARF patients with carditis observed valvar thickening defined as > 3.0mm in 75% of them [18], during a mean follow-up of 8.1 years, the leaflet thickening disappeared in 16 patients. Caldas et al., also using visual analysis, identified mitral leaflet thickening in the first ARF episode in 74% of patients who subsequently developed RHD in a five-year follow-up period [19]. Other investigations, either in autopsy [20] or echocardiographic series [21] established that the normal value for thickness of the mitral valve leaflets is between 2 and 3mm. The cut-off obtained in our study for mitral leaflet is higher, and in accordance to the proposed criteria of the World Heart Federation [4]. It also remained as the sole marker of prognosis either as a quantitative or categorical variable after multivariate analysis. It is a simple measurement that do not need advanced imaging techniques (e.g. harmonic imaging that should be avoided) [22].

Echocardiographic surveys focusing on identification of RHD in children of underdeveloped countries described mitral valve leaflet thickening as a marker of previous RHD among other findings [23–25]. However, our results demonstrated that thickening remained for up to 12 months in patients who later developed RHD, indicating the leaflet thickening as a persistent finding at least in the first year after an acute episode of ARF that could be of use for subsequent RHD epidemiological studies.

Mitral regurgitation, a frequent finding in ARF due to the inflammation of valvar and sub-valvar structures is commonly identified in RHD screening programs. It is a marker of the disease, but many machine parameters may influence measurements thus obtained [26, 27]. World Heart Federation criteria reinforce the need for adequately adjusting gain and other parameters in order to obtain jet measurements [4]. In our study, we kept machine and its parameters constant in the first year exam for all patients, reducing the chance of spurious measurements. Although it achieved a ROC of 0.75 it did not reached significance in the multivariate analysis models probably due to small sample size, especially in model 2 as a binary variable. Rémond et al., in accordance with our results, also did not observe the value of mitral regurgitation alone as a prognostic marker of RHD and only a combination of morphological and functional parameters of mitral valve structure and function [28]. Caldas et al. detected mitral regurgitation in 93% of patients with clinical carditis at baseline examination in a series of ARF patients and it persisted after 5 years in 59%, while in those without clinical carditis 37.9% had mild mitral regurgitation and at the final exam only 10.3% persisted with it. This finding reinforces our description that mitral regurgitation may not be a good prognostic marker for later development of RHD. Finally, a recent anatomopathological study demonstrated that the mitral valve has an increased thickness and active inflammation signs years after the acute rheumatic fever episode when patients were submitted to surgical replacement [29].

In our study, aortic regurgitation was equally distributed in both groups and jet dimension parameters were similar during the ARF episode. At 6-month follow-up no difference was observed either, making it a variable not relevant for multivariate analysis. Another probable reason for its exclusion was the concomitant presence of mitral involvement, making it a probable confounding variable. Our results also confirm the regression of aortic regurgitation as previously described in other series. As inflammation reduces, it is expected that valvar leakage would be reduced if the valve is not severely damaged. However, in a long-term follow-up even little damage may cause reappearance of leakage and manifest as RHD [30, 31].

### Limitations

Even though our study lacks a larger sample size, it comprises one of the largest ARF patients evaluated with Doppler echocardiography and with a long-term follow-up. Although previous ARF symptoms were not reported by our patients, we cannot definitely exclude this possibility as observed in other investigations. In addition, although every effort was made during follow-up to maintain antibiotic prophylaxis and to identify signs of new ARF episodes, it is not possible to be entirely sure that some recurrent episodes may have occurred in some patients. All measurements were made by one experienced cardiologist with expertise in echocardiography and revised by another but no intra or inter-variability was done. Since morphological measurements were linear and functional parameters had a standardized approach (fixed image parameters), variability was expected to be low.

## Conclusions

Our results demonstrate that Doppler echocardiography performed in patients with acute rheumatic fever can detect mitral valve leaflets thickening as a consistent prognostic marker for a more deleterious evolution to RHD during follow-up. Because rheumatic heart disease remains a major cause of morbidity and mortality in developing nations, these data have potentially important implications for delivery of effective secondary prevention.

## Abbreviations

ARF: Acute Rheumatic Fever
AV: atrioventricular
DEC: Doppler echocardiographic
LVEF: Left ventricular ejection fraction
LVOT: Left vetricularoutflow tract
EKG: Electrocardiogram
OR: Odds-ratio
ROC: Receiving operator curve
